# Stroke and Emerging Blood Biomarkers: A Clinical Prospective

**DOI:** 10.3390/neurolint14040065

**Published:** 2022-09-22

**Authors:** Aimilios Gkantzios, Dimitrios Tsiptsios, Stella Karatzetzou, Sofia Kitmeridou, Vaia Karapepera, Erasmia Giannakou, Penelope Vlotinou, Nikolaos Aggelousis, Konstantinos Vadikolias

**Affiliations:** 1 Neurology Department, Democritus University of Thrace, 68100 Alexandroupolis, Greece; 2 Department of Physical Education and Sport Science, Democritus University of Thrace, 69100 Komotini, Greece

**Keywords:** brain natriuretic peptide, fibrillary acidic protein, red cell distribution width, neutrophil-to-lymphocyte ratio, matrix metalloproteinase-9, aquaporin-4, stroke prognosis

## Abstract

Stroke constitutes the primary source of adult functional disability, exhibiting a paramount socioeconomic burden. Thus, it is of great importance that the prediction of stroke outcome be both prompt and accurate. Although modern neuroimaging and neurophysiological techniques are accessible, easily available blood biomarkers reflecting underlying stroke-related pathophysiological processes, including glial and/or neuronal death, neuroendocrine responses, inflammation, increased oxidative stress, blood–brain barrier disruption, endothelial dysfunction, and hemostasis, are required in order to facilitate stroke prognosis. A literature search of two databases (MEDLINE and Science Direct) was conducted in order to trace all relevant studies published between 1 January 2010 and 31 December 2021 that focused on the clinical utility of brain natriuretic peptide, glial fibrillary acidic protein, the red cell distribution width, the neutrophil-to-lymphocyte ratio, matrix metalloproteinase-9, and aquaporin-4 as prognostic tools in stroke survivors. Only full-text articles published in English were included. Twenty-eight articles were identified and are included in this review. All studied blood-derived biomarkers proved to be valuable prognostic tools poststroke, the clinical implementation of which may accurately predict the survivors’ functional outcomes, thus significantly enhancing the rehabilitation efficiency of stroke patients. Along with already utilized clinical, neurophysiological, and neuroimaging biomarkers, a blood-derived multi-biomarker panel is proposed as a reasonable approach to enhance the predictive power of stroke prognostic models.

## 1. Introduction

Stroke is not only the second leading cause of death worldwide but also the primary source of acquired disability within the adult population [1,2,3], resulting in chronically disabled survivors in up to 50% of all stroke cases, thus highlighting its paramount socioeconomic burden [2,4]. Taking into consideration the disease’s age-related character, with almost two thirds of patients being over the age of 65 [5], in conjunction with the continuous expansion of the world’s population and the significant improvement in life expectancy [2,6], stroke survivors’ numbers are expected to rise tremendously. As a result, the need for both prompt and accurate prognosis becomes crucial when taking into account the fact that the early identification of each patient’s recovery potential significantly facilitates decision making regarding the poststroke treatment strategy.

Nowadays, the implementation of advanced neuroimaging techniques as well as the application of highly effective reperfusion therapies during the period following a stroke enable the establishment of stroke diagnosis and promote the amelioration of stroke-related neurological deficits, thus enhancing the management of stroke patients and reducing the mortality risk. However, the aforementioned diagnostic and therapeutic methods are not widely accessible among stroke survivors, especially in developing countries. Apart from that, the fact that the neurological outcome poststroke appears to fluctuate considerably between a mild nondisabling type and a more severe type characterized by permanent disability or even death should be considered. Consequently, the utilization of blood biomarkers has emerged as a promising novel tool aimed at reliably forecasting functional outcomes poststroke [7,8,9].

According to the most recent definition, biomarkers represent physiological characteristics or biological substances that can be evaluated in an objective and reliable way and serve as indicators of either normal or pathological biological processes, risk factors, or pharmacologic responses to a therapeutic intervention. In order for a biomarker to be of clinical value, it should be characterized by both high sensitivity and specificity so that it has the potential to identify an existing disease while ruling out a disease that is not present [10,11,12]. Additionally, a biomarker aimed at optimizing the individualized management following stroke should exhibit some desirable characteristics and comply with specific requirements. More specifically, the ideal biomarker should be readily available, inexpensive, reproducibly and noninvasively obtained from easily available sources, acceptable to the patient, and easily interpretable by the clinician as well as cost-effective, providing information capable of affecting the therapeutic approach [4,13,14].

The role of stroke recovery biomarkers lies in enhancing the ability of clinicians to forecast the propensity for recovery following stroke by providing further insight into recovery processes. Ideally, a biomarker should guide therapeutic decision making in the setting of acute stroke, allocate health-care resources, and ultimately add to clinical care. Furthermore, the development of appropriate biomarkers might aid in promoting recovery and rehabilitation research by acting as surrogate markers and improving patient selection in clinical trials [15,16,17].

In order for a blood biomarker to be clinically relevant and accurately predict the long-term outcome following stroke, it should reflect underlying stroke-related pathophysiological processes, including glial and/or neuronal death, neuroendocrine responses, inflammation, increased oxidative stress, BBB disruption, endothelial dysfunction, and hemostasis [12]. Considering the variability in stroke patients’ clinical profiles and the complexity of the ischemic cascade as well as the heterogeneity of stroke phenotypes, it is obvious that a single biomarker may not be predictive for all stroke cases, thus making a multi-biomarker panel a potentially promising prognostic approach [12,18,19]. Among the wide range of promising candidate biomarkers that are currently under investigation, the present review focuses on six that meet the aforementioned desiderata: brain natriuretic peptide (BNP), as this is unquestionably the most well documented cardiac marker associated with stroke; glial fibrillary acidic protein (GFAP), the red cell distribution width (RDW), the neutrophil-to-lymphocyte ratio (NLR), and matrix metalloproteinase-9 (MMP9) due to the fact that these four are markers of inflammatory processes; and aquaporin-4 (AQP4), which tends to be a new trend in research into new biomarkers related to stroke prognosis. Emerging evidence suggests that the proposed blood-derived biomarkers hold the potential to enable accurate prognostication, significantly aid risk stratification, and efficiently promote the overall clinical management of stroke survivors, especially when concurrently utilized within a panel.

The prognostic utility of the aforementioned markers is not limited to an acute stroke setting. The management of a wide spectrum of medical/surgical diseases could be significantly enhanced by the implementation of appropriate blood markers. More specifically, the prognostication of various cardiovascular diseases’ outcomes, including acute coronary syndrome (ACS) and peripheral artery disease (PAD), might be promoted by the evaluation of NLR and MMP-9 by yielding additive information regarding a patient’s inflammatory status and atherosclerotic process [20,21]. Apart from that, NLR assessment may play a substantial role in the case of acute limb ischemia in terms of forecasting survival and the risk of amputation [22]. Strictly intertwined with inflammatory processes, the progression and postoperative complications of several malignancies as well as the clinical outcome of inflammatory diseases, such as systemic lupus erythematosus (SLE) or inflammatory bowel disease (IBD), could be predicted by the levels of GFAP [23], RDW [24], and NLR [25,26,27]. The prognostic significance of BNP and NLR is also evident in individuals presented with congestive heart failure [28,29].

Taking into account that the time period following a stroke can be considered a stress response, it is characterized by the activation of the sympathetic nervous system (SNS) and the hypothalamic–pituitary–adrenal (HPA) axis, resulting in elevated levels of many neuroendocrine biomarkers, BNP being among them. BNP constitutes a peptide-structured neurohormone whose role in hemodynamic regulation during the acute phase of ischemic stroke has been demonstrated in several clinical studies [30,31]. As far as GFAP is concerned, it is a highly brain-specific protein that cannot be detected in the blood plasma of healthy individuals, as it is not secreted under normal physiological conditions from human cells [32]. When a stroke occurs, both the destruction of glial brain cells and the disruption of the blood–brain barrier result in the release of great amounts of GFAP into the bloodstream, within minutes in case of ICH. In contrast, the cell destruction process evolves slower in patients presented with an ischemic stroke, thus leading to a delayed release of GFAP (typically between 6–12 h poststroke) and differentiating between the two main types of stroke [33,34].

With respect to a marker of anisocytosis known as RDW, the exact pathophysiological association between red blood cells (RBCs) and stroke remains unclear. Inflammation and oxidative stress (OS) may play an important role in determining the RDW in ischemic stroke. Inflammation reduces the survival rate of RBCs and inhibits the production or maturation of erythrocytes, thus leading to RBC damage. OS refers to the imbalance between in vivo oxidation and antioxidation, resulting in the oxidative impairment of RBC membranes, thus increasing RBC fragility, reducing erythrocytes’ life spans, and affecting the levels of RDW [35]. NLR, reflecting both innate (neutrophilic) and adaptive (lymphocytic) immune responses, has been widely studied. Increased levels of NLR indicate an imbalance between stroke-induced central inflammation and peripheral inflammation. Τhe clinical significance of NLR in stroke is attributed to the significant role inflammation plays, not only in stroke onset but also in the progression of brain injury and recovery [36,37,38,39].

Regarding MMPs, they constitute zinc- and calcium-dependent enzymes with proteolytic activity. More specifically, MMP-9 plays an important role in the development of various cerebrovascular diseases, including ischemic stroke and atherosclerosis, by degrading components of the extracellular matrix, leading to the weakening of the fibrous cap as well as activating proinflammatory cytokines and disrupting the function of the BBB [40,41]. As far as AQPs are concerned, they are specialized water channels expressed on heterogenous cell types. The presence of AQPs within the CNS facilitates bidirectional transmembrane water flow, mediating the development of cytotoxic edema during the acute stage of stroke and promoting edema resolution in the subacute stage of ischemia. The aforementioned process indicates a link between AQP-regulated water flux and neuroinflammation [42,43].

## 2. Materials and Methods

The Preferred Reporting Items for Systematic Reviews and Meta-analyses (PRISMA) checklist was used to guide this study. Our study’s methods were a priori designed.

### 2.1. Literature Search

A literature search of two databases (MEDLINE and Science Direct) was conducted by one investigator (AG) in order to trace all relevant studies published between 1 January 2010 and 31 December 2021 using the terms “blood biomarker” OR “blood test” AND “stroke outcome” OR “stroke prognosis” OR “stroke recovery” as keywords. In addition, the terms 1. “brain natriuretic peptide” OR “BNP”, 2. “glial fibrillary acidic protein” OR “GFAP”, 3. “red cell distribution width” OR “RDW”, 4. “neutrophil to lymphocyte ratio” OR “NLR’’, 5. “matrix metalloproteinase 9” OR “MMP-9”, and 6. “aquaporin-4” OR “AQP4” were used as secondary search criteria, each one separately with the aforementioned keywords. The retrieved articles were also hand-searched for any further potential eligible articles. Any disagreement regarding screening or the selection process was solved by a second investigator (KV) until a consensus was reached.

### 2.2. Eligibility Criteria

Only full-text original articles published in English were included. Secondary analyses, reviews, guidelines, meeting summaries, comments, unpublished abstracts, or studies conducted in animals were excluded. There were no restrictions on study design or sample characteristics.

### 2.3. Data Extraction

Data extraction was performed using a predefined data form created in Excel. We recorded the author, year of publication, biomarker, type of stroke, number of participants, time of blood sampling, scale(s) of stroke severity and of prognosis/clinical outcome and, finally, the main results of the study.

### 2.4. Data Analysis

No statistical analysis or meta-analysis was performed due to the high heterogeneity among the studies. Thus, the data were only descriptively analyzed.

## 3. Results

### 3.1. Database Searches

**Figure 1:**
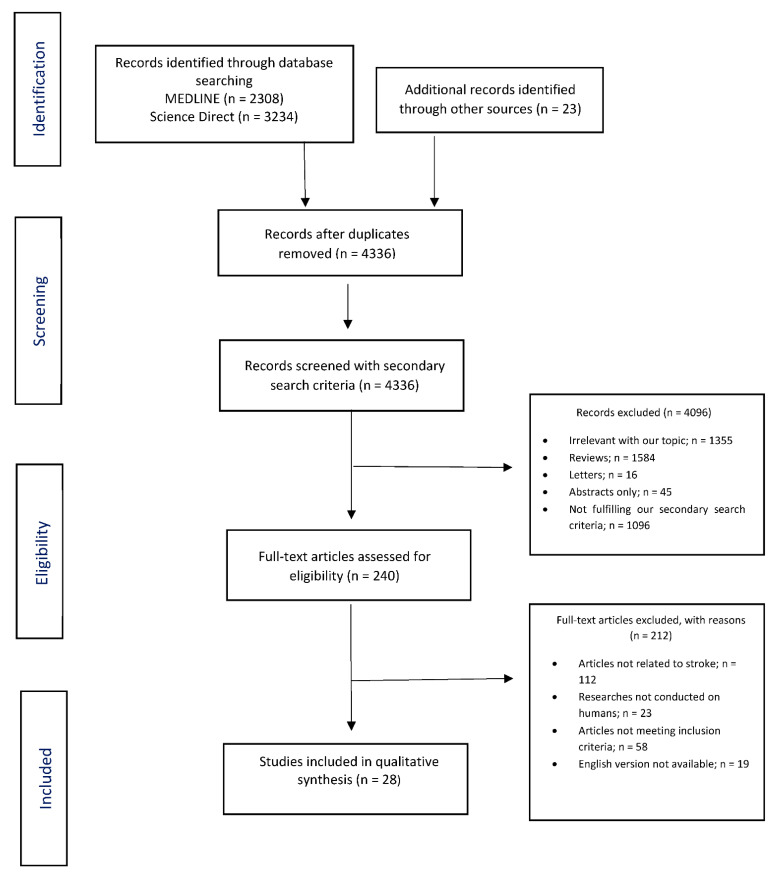
Study flow chart (PRISMA diagram).

Overall, 5565 records were retrieved from the database search. Duplicates and irrelevant studies were excluded. Then, 4336 records were screened with our secondary search criteria, and 240 full-text articles were selected as potentially eligible for our review. After screening the full text of the articles, 28 studies were ultimately eligible for inclusion (Figure 1).

### 3.2. Study Characteristics

Twenty-eight publications fulfilled our inclusion criteria. They were classified into six groups according to the type of biomarker examined. The first group comprised seven studies focusing on BNP and its association with stroke outcome. The second group included two studies dealing with the possible role GFAP could play in predicting each patient’s recovery potential, while the third group consisted of four studies examining the potential link between RDW and recovery following stroke. The fourth group included twelve studies investigating NLR as a potential prognostic biomarker poststroke. The fifth group included two studies that reported outcomes related to the utility of MMP-9 in the risk stratification of stroke survivors. Finally, the sixth group comprised one study dealing with the prognostic value of AQP-4 in the functional recovery of stroke patients. The characteristics of the included studies are presented in Table 1.

### 3.3. Study Design

In total, all studies included in this review were longitudinal. They were either retrospective or prospective cohorts.

### 3.4. Stroke Patient Groups

The total number of stroke patients included in all studies ranged from *n* = 42 [67,69] to *n* = 896 [44]. Across the 28 studies, 3 studies had disease sample sizes between 1 and 100 patients, 9 studies had disease sample sizes between 101 and 200, 6 studies had disease sample sizes between 201 and 300, 2 studies had disease sample sizes between 301 and 400, and 8 studies had disease sample sizes larger than 400 patients.

### 3.5. Reference Groups

Across the 28 studies, stroke patients were contrasted to demographically matched healthy individuals in only 4 studies, with the rest (24/28 studies) not including a healthy control group. None of the studies included a disease control group other than stroke patients.

### 3.6. Demographic and Clinical Profiles

The mean/median patient ages ranged from 56.0 years [66] to 76.3 years [48]. In total, 19 studies examined patients with IS, 7 studies examined patients with HS, and 2 studies examined patients with either IS or HS.

### 3.7. Time of Blood Sampling

In 20 studies, blood sampling was performed upon admission, in 4 studies blood sampling was performed in the first 24 h, in 1 study blood sampling was performed upon admission and 24 h after IV-TPA, in 1 study blood sampling was performed upon admission and within 3–7 days after, in 1 study blood sampling was performed 24 h after admission, and in 1 study blood samples were obtained before TEE.

### 3.8. Scales of Stroke Severity and Prognosis/Clinical Outcome

NIHSS and mRS were used in 20 studies. NIHSS was the only scale in two studies and mRS was exclusively used in three studies. In 3 out of 28 studies, combinations of scales of stroke severity and clinical outcome were used. More specifically, in one study, GCS, GOS, mRS, the Barthel Index, and the Allen scale were utilized; in one study, mRS and the Hunt Hess Scale were used; and finally, in one study, GCS, NIHSS, the ICH Score, the FUNC Score, and mRS were utilized.

## 4. Discussion

In recent years, interest in the research field of blood biomarkers has steadily increased. Up to today, numerous molecules have been identified, and a wide range of promising candidates have been suggested as stroke biomarkers. However, no individual blood biomarker has been proven to exhibit adequate sensitivity and specificity to be employed in everyday clinical practice [4,70]. The present review focuses on six blood biomarkers—BNP, GFAP, RDW, NLR, MMP-9, and AQP4—to elucidate their prognostic value poststroke.

With respect to BNP, since a high level of this specific blood biomarker might accompany a rather severe brain injury and is also associated with hemodynamic disturbances during the acute phase of stroke, its role as a prognostic indicator poststroke seems reasonable. For example, Montaner et al. [44] reported higher BNP plasma levels in stroke cases followed by either early neurological worsening or death, thus highlighting the positive correlation between BNP measures and both neurological deterioration and mortality after stroke. Interestingly, the prognostic value of BNP was applicable not only in ischemic stroke cases but also in patients presenting with brain hemorrhage. Despite the fact that BNP proved to be an independent indicator of poor outcome poststroke, the biomarker was not able to significantly enhance the predictive value of clinical prognostic models.

Maruyama et al. [45] found that BNP levels were strongly associated with infarct size as well as functional outcomes in cases of ischemic stroke. Moreover, patients presenting with cardioembolic stroke were characterized by significantly higher BNP levels compared to those that suffered from other ischemic stroke subtypes. Interestingly, a rise in circulating BNP levels was accompanied by an increase in the CHADS2 score as far as patients with atrial fibrillation (AF) were concerned, indicating a greater stroke risk within this specific population. The researchers concluded that natriuretic peptide levels may be considered useful biomarkers regarding the infarct size, the cardioembolic etiology of stroke event, and the long-term outcome as well as the stroke risk in AF patients. As far as acute ischemic stroke (AIS) outcomes in patients with nonvalvular atrial fibrillation (NVAF) are concerned, Maruyama et al. [48] reported that serum BNP levels were positively correlated both with the mRS score at 3 months and the CHADS2 score in NVAF patients, highlighting the role of BNP as an independent prognostic indicator poststroke. Similarly, in a study conducted by Otaki et al. [49] it was demonstrated that higher plasma BNP and N-terminal pro-brain natriuretic peptide (NT-proBNP) levels, both reflecting dysfunction of the left atrium and left atrial appendage, were superior in predicting cardiogenic stroke and subsequent major adverse cardiovascular and cerebrovascular events (MACCE) in stroke patients compared to either atrial natriuretic peptide (ANP) or high-sensitivity cardiac troponin T (hsTnT). The findings of the aforementioned study support the clinical utility of BNP and NT-proBNP as prognostic biomarkers of cardioembolism and future cerebrovascular events within a stroke population.

Apart from that, Tu et al. [46], in an attempt to provide insight into the linkage between baseline serum natriuretic peptide levels and short-term outcomes and mortality within 3 months poststroke, evaluated the prognostic value of an array of neuroendocrine biomarkers, including BNP, NT-proBNP, cortisol, and copeptin. The researchers found that the early measurement of selected neuroendocrine biomarkers was associated with stroke prognosis. More specifically, higher plasma BNP concentrations were coupled with an unfavorable functional outcome and increased mortality risk in stroke patients, thus highlighting its great prognostic potential. It is noteworthy that the inclusion of more than one readily measurable biomarker in a panel may significantly improve the prognostic accuracy, providing an accurate prediction of stroke outcome and death within 90 days. Furthermore, Nigro et al. [47] observed that BNP, but not sensitive cardiac troponin (scTnI), was able to independently predict death as a result of ischemic stroke or transient ischemic attack (TIA), while similar correlations were not demonstrated to exist between BNP levels and functional outcomes or stroke recurrence. As far as the stroke etiology is concerned, it was reported that the evaluation of BNP, in conjunction with the already known clinical variables, on admission appears to enhance the identification of patients with a cardioembolic source of stroke or TIA, thus improving the risk stratification and secondary prevention strategy of stroke survivors. Moreover, Chaudhuri et al. [30] explored the correlations between circulating plasma BNP levels and stroke subtypes and outcomes and showed that elevated BNP levels may serve as an independent predictor of acute ischemic stroke, as they were found in almost half of the stroke patients but only in a few control subjects. Regarding the prevalence of high BNP levels among stroke subtypes, the results of the study were in line with previous findings, reporting a significant association between elevated BNP levels and cardioembolic stroke, while noncardioembolic strokes were not demonstrated to be accompanied by a similar rise in BNP levels. The researchers also observed that stroke patients with higher plasma BNP levels presented with more severe strokes and were characterized by a higher risk of poor outcome and death.

With regards to GFAP as a prognostic biomarker poststroke, Xiong et al. [32], in an attempt to investigate the utility of GFAP testing in distinguishing between intracerebral hemorrhage (ICH) and ischemic stroke (IS), studied both ICH and IS patients and evaluated their GFAP levels early after stroke onset. The researchers found that serum GFAP concentration was significantly higher within the ICH group compared to the IS group 2–6 h after symptoms onset, thus highlighting the role of GFAP as an efficient tool in differentiating between the types of stroke. Moreover, GFAP levels were demonstrated to be positively correlated with hemorrhage volume and neurological deficit, as ICH patients suffering from larger and more severe hemorrhages were characterized by elevated GFAP levels. As far as the functional outcome is concerned, the GFAP test seems to be able to predict short-term outcomes, as an increase in GFAP concentration is significantly associated with an unfavorable prognosis regarding ICH patients. It is noteworthy that similar correlations between GFAP levels and infarct size, stroke severity, and outcome were not observed in the IS group, a finding that was attributed to the different stroke evolution processes between brain hemorrhage and ischemia. In contrast, Liu et al. [50] concluded that plasma GFAP levels on admission have the potential to accurately predict the long-term functional outcome post-AIS, significantly enhancing the prognostic value of NIHSS. More specifically, it was shown that serum GFAP concentration early after ischemic stroke onset correlates well with ischemic lesion size and clinical severity among stroke survivors and appears to be a useful biomarker of poor prognosis in the case of AIS.

As far as the prognostic utility of RDW in AIS, Kim et al. [51] investigated the potential linkage between RDW measured early after stroke onset and long-term functional outcome and observed that increased RDW was strongly associated with both a poor prognosis and mortality, as RDW levels were significantly higher in patients who exhibited poor functional outcomes at 3 months as well as those who died within 3 months or during the first year poststroke. As a result, RDW may be utilized as a prognostic biomarker for the prediction of functional outcomes and survival in patients with acute ischemic stroke. Moreover, Ye et al. [52] concluded that the baseline assessment of RDW was positively correlated with the one-year mortality risk in patients with acute ischemic stroke who were treated with intravenous thrombolysis (IVT), thus indicating a potential role of RDW as an independent biomarker of all-cause death during the first year in stroke patients after IVT. Unlike mortality risk, a significant association between RDW levels and functional outcome in stroke patients undergoing IVT was not demonstrated. Thus, RDW before thrombolysis may serve as a useful prognostic tool regarding one-year mortality rather than as a predictor of clinical outcome poststroke. Similarly, Cong et al. [53] showed that the RDW test was predictive of the 90-day clinical outcome after IVT, as half of the patients with elevated RDW values exhibited an unfavorable functional prognosis, whereas only one fourth of the patients with normal RDW levels had poor clinical outcomes. Apart from that, Cui et al. [54] explored the prognostic utility of RDW in patients with intracerebral hemorrhage (ICH) and observed that a high RDW value was strongly associated with 30-day mortality and an unfavorable prognosis in ICH cases. Interestingly, the researchers established a prognostic nomogram incorporating RDW, aimed at determining the 30-day functional prognosis in ICH patients, with high predictive accuracy.

As far as the prognostic value of NLR following a stroke is concerned, Brooks et al. [55] reported a significant correlation between the NLR value and stroke prognosis after endovascular recanalization therapy, as elevated NLR levels were coupled with poor outcomes and death, while a rather normal NLR value appeared to be predictive of functional independence at 90 days poststroke. As a reflection of immune dysregulation among stroke patients, increased NLR may act as a useful prognostic tool aimed at identifying which patients will exhibit favorable outcomes and will subsequently benefit the most from endovascular interventions. It is noteworthy that an interaction between NLR and age was also found, and more specifically, in patients over 80, NLR was not able to accurately predict stroke outcomes, thus suggesting the limited utility of the biomarker in older patients. Moreover, Malhorta et al. [58] aimed at exploring the efficacy and safety profile of IVT in AIS patients and showed that lower NLR values were significantly associated with both favorable functional outcomes and functional independence at three months poststroke, while elevated NLR levels on admission were independently predictive of higher mortality risk at three months. The researchers concluded that the early assessment of NLR counts might provide clinically useful prognostic information when evaluating stroke patients as candidates for recanalization therapies, thus facilitating the decision-making process after a stroke insult.

Apart from that, in a study conducted by Pektezel et al. [60], it was demonstrated that the evaluation of the NLR value in stroke patients undergoing IVT is of key importance regarding thrombolysis effectiveness and the long-term functional prognosis as well as symptomatic intracranial hemorrhage occurrence. It should be noted that the timing of NLR assessment (on admission vs. 24 h post-treatment) appears to determine the prognostic utility of the biomarker. More specifically, elevated post-IVT NLR levels, reflecting a stroke-associated stress response accompanied by both lymphopenia and neutrophilia, was found to be associated with a remarkable resistance in thrombolytic treatment, a high risk of IVT-induced intracranial hemorrhage, and an unfavorable prognosis at three months poststroke. On the contrary, the pretreatment NLR count was not able to serve as an independent predictor of IVT responsiveness and prognosis in acute ischemic stroke survivors. Additionally, Topcuoglu et al. [63], in an attempt to establish clinically useful NLR thresholds, reported a moderate utility of the NLR value as a predictor of IVT-associated hemorrhagic complications, thus pointing out the significant role of NLR in parallel with other variants in evaluating the risk of hemorrhagic transformation in stroke patients treated with IVT and, potentially, in determining the overall functional prognosis. As far as the predictive ability of NLR in cases of large vessel occlusion (LVO) stroke after endovascular thrombectomy (EVT) is concerned, Aly et al. [62] observed a strong correlation between both the 3–7-day follow-up NLR value and temporal NLR changes with reperfusion status and, subsequently, the clinical outcome at 3 months poststroke. A successful recanalization of the occluded artery as well as a good 90-day functional outcome were significantly predicted by lower follow-up NLR counts and less dynamic NLR changes, whereas elevated NLR levels and more temporal NLR changes were found to be positively associated with the occurrence of symptomatic intracranial hemorrhage and mortality risk. The admission NLR in patients that were being considered for EVT was not found to be significantly related with clinical outcomes. Similarly, Chen et al. [64] explored the relationship between NLR levels and clinical outcomes in patients suffering from an LVO stroke that underwent EVT. The researchers concluded that higher NLR values were followed by an unfavorable prognosis after the endovascular recanalization therapy, highlighting the role of NLR in prognosticating EVT’s outcomes. Regarding the bleeding risk after EVT in stroke patients, Pikija et al. [59] investigated the occurrence of hemorrhagic complications post-EVT and found that the development of intracranial hemorrhage as an adverse event post-EVT was independently predicted by higher admission NLR counts and was accompanied by increased 3-month mortality.

With respect to the prognostic value of the systemic inflammation status reflected in the NLR value in acute intracerebral hemorrhage patients, Sun et al. [56] first reported a positive correlation between NLR at admission and both the hematoma volume and the baseline stroke severity assessed with NIHSS in ICH patients. It should also be noted that NLR was not found to be significantly associated with the 3-month clinical outcome. Furthermore, Tao et al. [57], having investigated the potential linkage between the inflammation marker, NLR, and the 90-day outcome after ICH, demonstrated the biomarker’s remarkable predictive value regarding mortality and functional outcomes poststroke. Similarly, Menon et al. [65] evaluated the influence of the NLR biomarker on ICH course in terms of mortality and functional outcomes and found that NLR values were higher in subgroups of patients that exhibited early neurological deterioration or died at 30 days and 90 days poststroke. In line with previous studies, elevated admission levels of NLR were associated with poor outcomes, as patients with unfavorable outcomes presented with significantly higher NLR values in comparison with patients that were characterized by good functional outcomes. Additionally, Fonseca et al. [61] aimed at elucidating the effect of NLR values in the clinical outcomes of ICH patients and assessed the potential mechanisms mediating neurological deterioration after ICH and their correlations with admission NLR. The researchers observed a strong association between high baseline NLR counts and an unfavorable prognosis that was attributed to the early development of perihematomal edema rather than the hematoma expansion itself. Apart from that, Chang et al. [66], in an attempt to explore the role of NLR as a prognostic indicator in patients with aneurysmal subarachnoid hemorrhage (aSAH), showed that elevated NLR has the potential to independently predict a poor functional outcome at discharge within a population of aSAH patients, indicating the responsible underlying inflammatory pathophysiological mechanisms.

Regarding the prognostic utility of MMP-9 after stroke, in a study conducted by Sapojnikova et al. [67] it was reported that MMP-9 activation was significantly associated with a high MMP-9 plasma concentration in ischemic stroke patients, thus highlighting the fact that the levels of the active form of this proteolytic enzyme are elevated poststroke and play an important role in brain ischemia evolution. It is of great interest that the MMP-9 concentration only increased in patients that presented with a total anterior circulation infarction. According to the study’s findings, increased serum MMP-9 levels were strongly correlated with an unfavorable functional outcome, and subsequently the early measurement of circulating MMP-9 may serve as an effective indicator of neurological outcome following acute ischemic stroke. Apart from that, Zhong et al. [68], in an attempt to further explore the implication of MMP-9 in stroke prognosis, concluded that the serum MMP-9 concentration in the acute phase of ischemic stroke was positively associated with the risk of mortality and major disability at 3 months, supporting the use of MMP-9 as a potential prognostic biomarker poststroke. Interestingly, the associations observed between elevated levels of MMP-9 and higher risks of death and major disability among stroke patients were characterized by a dose–response pattern.

With respect to the potential link between AQP-4 and stroke outcomes, Ramiro et al. [69], having studied stroke patients treated with IVT, observed an increase in the AQP-4 detected in the circulation early after the stoke event, reflecting the stroke-mediated upregulation of AQP-4 levels in the brain within the first hours poststroke. Additionally, it was demonstrated that patients with high baseline AQP-4 levels showed an early neurological amelioration, both at 48 h poststroke and at discharge, thus pointing out the great potential of AQP-4 in predicting the clinical outcomes of stroke survivors treated with intravenous thrombolysis. As a result, the assessment of circulating AQP-4 levels during the acute phase of stroke seems to be able to facilitate stroke prognosis and subsequently optimize patients’ clinical management and final neurological outcomes.

### Limitations

We acknowledge that our study has some limitations that need to be considered. First, the limited sample size may impede the generalizability of the study’s results and, therefore, lead to bias. Before broader implementation, further larger-scale studies are required to validate our findings. Another potential limitation is the fact that the influence of the time of biomarker level measurement was not considered, and the role of treatment, possibly affecting the biomarkers’ concentrations, was not analyzed in the present study. The aforementioned limitations must be addressed in order to obtain more reliable results.

## 5. Conclusions

Overall, the results of the present review are indicative of a link between the circulating levels of BNP, GFAP, RDW, NLR, MMP-9, and AQP4 following stroke and clinical outcome prognostication. Being able to yield additive information regarding stroke clinical severity, functional outcome, and treatment efficiency as well as to differentiate between stroke survivors with favorable prognoses and those individuals with poor recovery propensity poststroke, the aforementioned blood-derived biomarkers are emerging as novel aids in the acute stroke setting. Moreover, given the fact that stroke represents a rather heterogenous syndrome, a combined panel incorporating BNP, GFAP, RDW, NLR, MMP-9, and AQP4 might represent a promising approach, both in research and in clinical practice, minimizing the effect of any individual biomarker. Implementing such a panel in a reasonable and cost-effective way may be a valuable tool following stroke.

## Figures and Tables

**Table 1:** Basic characteristics of the 28 included studies.

	Authors, Year of Publication	Biomarker	Type of Study	Type of Stroke	Number of Participants/Mean Age	Time of Blood Sampling	Scale of Stroke Severity and Prognosis/Clinical Outcome	Cut-Off Values; (Specificity); [Sensitivity]	Main Results
1.	Montaner et al. 2012 [44]	BNP	Longitudinal	IS and HS	896 patients/72 (SD 12)	Upon admission (within 24 h from symptom onset)	NIHSS (on admission and at discharge)	For neurological deterioration: BNP > 56.7 ng/L; (47.9%); [68.8%] For death after stroke: BNP > 65.3 ng/L; (51.2%); [73.5%]	For both IS and HS, elevated BNP levels were associated with early neurological deterioration and mortality.
2.	Maruyama et al. 2013 [45]	BNP	Longitudinal	IS	231 patients/71 ± 12	Upon admission	NIHSS (on admission), mRS (at discharge or 2 months after stroke onset)	Types of IS: SVO: 29 pg/mL (13–91); (N.A.); [N.A.] LAA: 54 pg/mL (30–101); (N.A.); [N.A.] CE: 202 pg/mL (120–385); (N.A.); [N.A.] Other: 12 pg/mL (7–57); (N.A.); [N.A.]	High BNP levels might serve as a useful biomarker to predict cardioembolism (CE) and its clinical outcome as well as the size of cerebral infarct and the risk for stroke in AF patients.
3.	Tu et al. 2013 [46]	BNP	Longitudinal	IS	189 patients/66 (IQR 58–75)	The first morning after admission	NIHSS (on admission and on day 90), mRS (at discharge or within 90 days)	100 pg/mL; (N.A.); [N.A.]	Increased BNP levels were associated with IS clinical severity and unfavorable short-term outcomes.
4.	Nigro et al. 2014 [47]	BNP	Longitudinal	IS and TIA	441 patients/74.6 (IQR 62.6–81.9)	Upon admission (within 72 h from symptom onset)	NIHSS (on admission), mRS (3 and 12 months after stroke onset)	N.A.	Increased BNP levels can be used to predict unfavorable clinical outcomes and mortality within 90 days as well as in the first year after stroke. In addition, BNP levels were higher in patients with a cardioembolic stroke or TIA.
5.	Chaudhuri et al. 2015 [30]	BNP	Longitudinal	IS	270 patients, 110 healthy controls/patients: 59 (21–87), controls: 58 (23–85)	Upon admission	NIHSS and mRS (on admission and 3 months after stroke onset)	89 pg/mL; (62.2%); [83.9%]	A significant association was noticed between elevated BNP levels and cardioembolic stroke.
6.	Maruyama et al. 2017 [48]	BNP	Longitudinal	IS	168 NVAFpatients with cardioembolic stroke, 157 were eligible for analysis/76.3 ± 10.2	Upon admission	NIHSS (on admission), mRS (3 months after stroke onset)	Poor functional outcome: BNP > 115 pg/mL; (N.A.); [N.A.]	A positive correlation between BNP levels on admission and the mRS score at 3 months in patients with NVAF after AIS was detected.
7.	Otaki et al. 2019 [49]	BNP	Longitudinal	IS	282 patients, 138 controls/patients: 71 ± 12, controls: 67 ± 13	Blood samples were obtained before TEE (transesophageal echocardiography)	NIHSS	For cardiogenic stroke: 58.2 pg/mL; (N.A.); [N.A.] For MACCE: 73.4 pg/mL; (N.A.); [N.A.]	BNP can be used as a biomarker to predict cardiogenic stroke.
8.	Xiong et al. 2015 [32]	GFAP	Longitudinal	HS (ICH) and IS	43 ICH and 65 IS patients/ICH patients: 68.7 ± 11.2, IS patients: 70.9 ± 9.6	Upon admission	NIHSS (on admission), mRS (3 months after stroke onset)	Differentiation between ICH and IS: 0.7 ng/mL; (76.9%); [86.0%] Poor functional outcome: 1.04 ng/mL; (80.0%); [95.7%]	Differences in serum concentrations of GFAP between ICH and IS can be used to differentiate strokes. In patients with ICH, GFAP levels were higher.
9.	Liu et al. 2018 [50]	GFAP	Longitudinal	IS	286 patients/Q1 3: 61 (50–72), Q4: 67 (56–78)	On the first day of admission	NIHSS (on admission), mRS (one year after stroke onset)	0.25 [0.16–0.34] ng/mL in patients with moderate-to-high clinical severity; (N.A.); [N.A.]	GFAP levels on admission may predict clinical and functional outcomes after IS.
10.	Kim et al. 2012 [51]	RDW	Longitudinal	IS	847/65.88 ± 12.45	Upon admission	NIHSS (on admission), mRS (3 months after stroke onset)	Poor outcome: RDW > 13.37 ± 1.41; (N.A.); [N.A.] Mortality at 3 months: RDW > 14.04 ± 1.71; (N.A.); [N.A.]	Higher values of RDW were associated with poor functional outcomes and higher mortality.
11.	Ye et al. 2020 [52]	RDW	Longitudinal	IS	480/71 (IQR 16)	Upon admission	NIHSS (on admission and at discharge), mRS (at discharge and one year after stroke onset)	14.65; (88.3%); [42%]	RDW before thrombolysis could be used as a predictor of one-year mortality but not for stroke severity prognostication.
12.	Cong et al. 2020 [53]	RDW	Longitudinal	IS	196/64.22 ± 12.45	Upon admission	NIHSS (on admission, 24 h after stroke onset, and at discharge), mRS (on admission, at discharge, and 3 months after stroke onset)	Poor prognosis ≥ 13.15; (60.7%); [64.0%]	An elevated RDW value was associated with poor prognosis in patients who received rtPA thrombolysis.
13.	Cui et al. 2020 [54]	RDW	Longitudinal	HS (ICH)	235/64.6 ± 14.5	Upon admission	mRS (30 days after stroke onset)	Nonsurvivors: 14.7 ± 1.2; (N.A.); [N.A.] Unfavorable outcome: 14.2 ± 1.3; (N.A.); [N.A.]	High RDW values were correlated with poor clinical outcomes in patients with ICH.
14.	Brooks et al. 2014 [55]	NLR	Longitudinal	IS	116/67 (18–93)	Upon admission	NIHSS (on admission), mRS (3 months after stroke onset)	Poor outcome: NLR ≥ 5.9; (N.A.); [N.A.] Functional independence: NLR < 3.2; (N.A.); [N.A.]	In patients with IS who underwent endovascular treatment, NLR > 5.9 predicted poor outcomes and death and NLR < 3.2 predicted an outcome of functional independence.
15.	Sun et al. 2016 [56]	NLR	Longitudinal	HS (ICH)	352/64.2 ± 13.8	Upon admission	NIHSS (on admission), mRS (3 months after stroke onset)	NLR ≥ 7.85; (N.A.); [N.A.]	Patients with higher admission NLR values had larger hematoma volumes and higher baseline NIHSS scores.
16.	Tao et al. 2016 [57]	NLR	Longitudinal	HS (ICH)	336/58.5 ± 13.0	Upon admission	mRS (3 months after stroke onset)	For predicting poor 90-day outcome: NLR > 6.28; (57.4%); [73.3%] For predicting 90-day mortality: NLR > 6.62; (N.A.); [N.A.]	Elevated levels of NLR can predict poor 90-day outcomes after ICH.
17.	Malhotra et al. 2018 [58]	NLR	Longitudinal	IS	657/64 ± 14.4	Within 12 h from admission	NIHSS (on admission), mRS (3 months after stroke onset)	For 3-month favorable functional outcome and functional independence: NLR < 2.2; (63.1%); [51.4%]	In AIS patients treated with IVT, lower NLR levels were associated with favorable clinical outcomes at 3 months.
18.	Pikija et al. 2018 [59]	NLR	Longitudinal	HS (ICH)	187/74 (IQR 60–81)	Upon admission	NIHSS (on admission and at discharge), mRS (3 months after stroke onset)	3.89; (73.0%); [67.0%]	Patients who developed ICH after EVT had higher admission NLR values.
19.	Pektezel et al. 2019 [60]	NLR	Longitudinal	IS	142/69 ± 13	Upon admission and after 24 h	NIHSS (on admission, at 24 h after IV tPA, and at discharge), mRS (3 months after stroke onset)	NLR ≤ 3.2 indicates IV tPA effectiveness; (84%); [48%] NLR ≤ 3.6 indicates favorable prognosis; (73%); [65%] NLR ≤ 7.4 indicates absence of symptomatic cerebral hemorrhage; (76%); [100%]	Increased NLR values during the first 24 h were associated with poor prognosis. Pretreatment NLR values seemed to have no connection with the IV tPA response.
20.	Fonseca et al. 2019 [61]	NLR	Longitudinal	HS (ICH)	135/73 (64–80)	Upon admission	GCS (on admission), NIHSS (on admission), ICH score (on admission), FUNC score (on admission), mRS (3 months after stroke onset)	Independence at 90 days (mRS 0 to 2): NLR < 2.3; (N.A.); [N.A.] Mortality at 30 days: NLR > 4.4; (N.A.); [N.A.] Significant early cerebral edema: NLR > 8.06; (N.A.); [N.A.]	Higher NLR values at admission were associated with unfavorable functional outcomes.
21.	Aly et al. 2021 [62]	NLR	Longitudinal	IS	142/70 ± 16	Upon admission, (within 3–7 days)	NIHSS (on admission), mRS (3 months after stroke onset)	Favorable outcome: follow-up NLR at 3–7 days < 5.3; (68.0%); [76.0%]	Lower follow-up NLR at 3–7 days was associated with successful reperfusion and positive clinical outcomes.
22.	Topcuoglu et al. 2021 [63]	NLR	Longitudinal	IS	165/70 ± 14	Upon admission and 24 h after IV tPA	NIHSS (on admission and at 24 h after IV tPA), mRS (3 months after stroke onset)	For symptomatic PH2-type hemorrhagic transformation: NLR > 5.65; (65.7%); [71.3%]	Patients who responded well to IV tPA had lower NLR values. On the other hand, those patients who developed symptomatic hemorrhagic transformation after IV tPA had higher pretreatment NLR values.
23.	Chen et al. 2021 [64]	NLR	Longitudinal	IS	257 AIS patients who underwent EVT/63.2 ± 12.6	Upon admission	NIHSS (on admission, within 72 h), mRS (3 months after stroke onset)	N.A.	Increased levels of NLR may be associated with unfavorable clinical outcomes in stroke patients who underwent EVT.
24.	Menon et al. 2021 [65]	NLR	Longitudinal	HS (ICH)	851/58.09 ± 12.85	Upon admission	mRS (3 months after stroke onset)	NLR > 8.2; (N.A.); [N.A.]	NLR above the cutoff of 8.2 at admission was associated with unfavorable functional outcomes and high mortality.
25.	Chang et al. 2021 [66]	NLR	Longitudinal	HS (SAH)	474/56 ± 16	Upon admission	mRS (at discharge), Hunt Hess Scale (at discharge)	Poor functional outcome at discharge: NLR > 6.48; (N.A.); [N.A.]	Higher NLR values at admission corresponded to poor functional outcomes in aSAH patients.
26.	Sapojnikova et al. 2014 [67]	MMP-9	Longitudinal	IS	42 patients, 32 healthy controls/patients: 69 ± 15, healthy controls: 63 ± 20	Upon admission	GCS (on admission), GOS (at discharge, one month after IS), Barthel index, mRS, and Allen scale	N.A.	A linear correlation was detected between highlevels of MMP-9 and poor functional outcomes.
27.	Zhong et al. 2017 [68]	MMP-9	Longitudinal	IS	767/62.4 ± 10.8	Within 24 h of hospital admission	NIHSS (on admission). mRS (3 months after stroke onset)	812.2 ng/mL; (N.A.); [N.A.]	Elevated MMP-9 levels in the acute phase of ischemic stroke were accompanied by increased risks of mortality and major disability.
28.	Ramiro et al. 2019 [69]	AQP4	Longitudinal	IS	42 t-PA-treated patients, 13 healthy controls/patients: 74 (66–78), controls: 76 (70–83)	Upon admission	NIHSS (on admission, at 48 h, and at discharge), mRS (3 months after stroke onset)	cut-off point for AQP4 associated with patients’ neurological improvement 48 h after stroke: 2.52 ng/mL; (89.9%); [54.5%] cut-off point for AQP4 that was associated with neurological improvement by the time of hospital discharge: 1.72 ng/mL; (76.9%); [58.6%]	Lower circulating AQP4 levels were associated with early neurological improvement. In addition, an inverse correlation was found between AQP4 values and the infarct size.

**Abbreviations.** AF: atrial fibrillation, aSAH: aneurysmal subarachnoid hemorrhage, AQP4: aquaporin-4, BNP: brain natriuretic peptide, CE: cardioembolism, EVT: endovascular thrombectomy, FUNC: Functional Outcome in Patients with Primary Intracerebral Hemorrhage, GCS: Glasgow Coma Scale, GFAP: glial fibrillary acidic protein, GOS: Glasgow Outcome Scale, HS: hemorrhagic stroke, ICH: intracerebral hemorrhage, IQR: interquartile ranges, IS: ischemic stroke, IVT: intravenous thrombolysis, LAA: large artery atherosclerosis, MACCE: major adverse cardiovascular and cerebrovascular events, MMP-9: matrix metalloproteinase-9, mRS: modified Rankin Scale, N.A.: not applicable, NIHSS: National Institutes of Health Stroke Scale, NLR: neutrophil-to-lymphocyte ratio, NVAF: nonvalvular atrial fibrillation, RDW: red cell distribution width, SAH: subarachnoid hemorrhage, SVO: small vessel occlusion, TIA: transient ischemic attack.

## Data Availability

All data discussed within this manuscript are available on PubMed.
